# Methamphetamine Shows Different Joint Toxicity for Different Types of Microplastics on Zebrafish Larvae by Mediating Oxidative Stress

**DOI:** 10.3390/toxics12010009

**Published:** 2023-12-22

**Authors:** Jindong Xu, Wenqi Yang, Dongyi Wang, Zhenglu Wang, Chuang Liu, Jiana Li

**Affiliations:** 1 College of Oceanography, Hohai University, Nanjing 210098, China; aukuma20@gmail.com (J.X.); eliomurphy8@gmail.com (W.Y.); wdynn@foxmail.com (D.W.); huangliu2020@hhu.edu.cn (C.L.); 2 West China School of Public Health and West China Fourth Hospital, Sichuan University, Chengdu 610041, China; m18018567955@163.com; 3 Ningbo Academy of Ecological and Environmental Sciences, Ningbo 315000, China

**Keywords:** microplastics, methamphetamine, joint toxicity, behavioral functions, oxidative stress

## Abstract

The coexistence of polystyrene (PS) and polypropylene (PVC) microplastics (MPs) and methamphetamine (METH) in aquatic systems is evident. However, the joint toxicity is unclear. Here, zebrafish larvae were exposed to single PS and PVC MPs (20 mg L^−1^) and combined with METH (250 and 500 μg L^−1^) for 10 days. The results indicated that acute exposure to PS and PVC MPs induced lethal effects on zebrafish larvae (10–20%). Treatment with MPs markedly suppressed the locomotion of zebrafish, showing as the lengthy immobility (51–74%) and lower velocity (0.09–0.55 cm s^−1^) compared with the control (1.07 cm s^−1^). Meanwhile, histopathological analysis revealed pronounced depositions of MPs particles in fish’s intestinal tract, triggering inflammatory responses (histological scores: 1.6–2.0). In the coexposure groups, obviously inflammatory responses were found. Furthermore, the up-regulations of the genes involved in the oxidative kinase gene and inflammation related genes implied that oxidative stress triggered by MPs on zebrafish larvae might be responsible for the mortality and locomotion retardant. The antagonistic and stimulatory effects of METH on the expression changes of genes found in PVC and PS groups implied the contrary combined toxicity of PS/PVC MPs and METH. This study for the first time estimated the different toxicity of PS and PVC MPs on fish and the joint effects with METH at high environmental levels. The results suggested PS showed stronger toxicity than PVC for fish larvae. The addition of METH stimulated the effects of PS but antagonized the effects of PVC, promoting control strategy development on MPs and METH in aquatic environments.

## 1. Introduction

In conjunction with the growth of the plastic manufacturing industry, microplastics (MPs)—small particles derived from plastic waste—have emerged as environmental pollutants in aquatic systems [1]. Statistically, the total yield of plastic production in 2018 was 359 million tons worldwide, with 5–10% of that drained into the ocean [2]. Over time, plastic waste breaks down into smaller particles through the action of microorganisms, light, and hydraulic erosion, with particles smaller than 5 mm in diameter classified as MPs [3]. According to the monitoring results, the concentrations of MPs in the Yangtze Estuary and Taihu Lake were 10,200 items m^−3^ and 25,800 items m^−3^, respectively [4,5]. In the Rhine River, 892,777 MP items per square kilometer were detected [6]. However, it is crucial to emphasize that these measurements mainly pertain to MPs with larger particle sizes. A survey revealed that, for MPs with smaller sizes, 1–10 μm MPs constitute approximately 80% of the pollution found in water bodies [7]. Among them, polystyrene (PS), polyethylene (PE), and polypropylene (PP) are the predominant MPs and have been frequently detected in marine animals, such as mussels and fishes [8]. When ingested by organisms through feeding, MPs can induce a range of adverse effects, including oxidative stress, behavioral dysfunctions, immunological responses, and morphological damage [9,10,11,12]. Even in the absence of ingestion, MPs may still pose a threat to organisms. Previous studies have shown that MPs can adsorb onto the envelope of zebrafish embryos after short-term exposure, leading to aberrant behaviors in the larvae [10]. Hence, the threat of MPs to the environment should be considered. Furthermore, the outcomes of MPs may vary depending on their characteristics, such as polymer types, additives, and surface properties, leading to heterogeneity in their effects [13,14]. The underlying mechanisms of such opposite effects are still unclear.

In China, PS is a major contributor to MPs pollution in water, with an annual production of up to 1.8 million tons [15]. Indeed, previous research has provided evidence of the toxicity of larger-sized PS, typically ranging from 75 to 200 μm, on zooplankton organisms [16]. It is well known that the average size of PS MPs in surface water falls within the range of 10–20 μm [17]. However, there is a lack of comprehensive assessment regarding the potential impairments specifically posed by MPs of smaller sizes. The existing studies indicate that surface modification and pH can influence the aggregation of nano-sized PS MPs in aquatic environments. Furthermore, it has been confirmed that these factors can cause damage to the digestive tract of large crayfish [18]. However, at a concentration of 1 mg/L, no significant toxicity was observed in zebrafish [19]. Additionally, it is important to note that PVC is another commonly used plastic that may have toxic effects on organisms due to its functional groups and additives [20]. Previous studies have shown that PVC can inhibit the increase in body weight and length of *Cyprinus carpio* var. larvae and cause oxidative stress damage and hepatocyte vacuolation [21]. Another study showed that PVC can inhibit the expression of genes related to heart development in zebrafish larvae, resulting in the weakening of heart function [22]. Nevertheless, the adverse effects on fishes lack more evidence.

Moreover, MPs feature the potential to act as chemical vectors for some organic chemicals in aquatic environments due to their large specific surface area and high absorbability [23]. This finding derives a new scenario for coexposure to MPs and organic contaminants, which may lead to joint effects on organisms. When ingested by organisms, coexposure to MPs and other environmental contaminants could enhance the bioavailability and adverse effects on biota compared to monomer MPs exposure, including increased accumulation of organic pollutants, abnormal glycolipid metabolism, hepatotoxicity, and endocrine disruption [24,25,26]. However, the combined toxicity of MPs and organic pollutants is extremely complex. For example, the presence of PS MPs significantly decreased the harm to mussels caused by PAHs [27] but increased the toxicity of triclosan to marine copepods [28]. Meanwhile, some studies have shown that the toxicity of PVC and DEHP can be antagonized, and it is believed that PVC can reduce its toxicity by increasing the adsorption of DEHP [29]. The underlying mechanisms of such opposite effects are still unclear. Nevertheless, it can be ascertained that variations in the adsorption capacity of different types of MPs and organic pollutants exist [30,31]. The adsorption relationship between MPs and organic pollutants can also be influenced by factors such as changes in pH, the presence of cations, and the aging degree of water [30,31]. These factors are considered to be contributors to the observed differences in the joint effects of MPs and organic pollutants. Further research is required to fully elucidate the intricate mechanisms underlying these phenomena [32,33].

As emerging organic pollutants, illicit drugs are of great concern due to their ubiquity, teratogenic potency, and neurotoxicity [22,34]. Methamphetamine (METH) is the primary illicit drug widely detected in the surface water of China, with the highest concentrations up to 405 ng L^−1^ [35]. In addition, METH is also widely detected worldwide. In the Nepean River of New South Wales in Australia, the detected concentration is up to 25.25 μg L^−1^ [36], and in the Llobregat basin in Spain, the detected concentration is 50 ng L^−1^ [37]. Previous studies found that exposure to METH at low environmental levels could induce obvious malformation of zebrafish larvae [12,38]. Meanwhile, another article shows that exposure to METH can lead to behavioral abnormalities and certain reproductive toxicity in guppies [22]. In addition, studies have shown that METH at environmental levels can lead to wild brown trout addiction and change habitat preferences, which may have adverse consequences at the population level [39]. The persistent occurrence of METH in water might result from its interaction with MPs. Qu et al. reported that METH is adsorbed by PS MPs and promotes their bioavailability [40], suggesting that it is warranted to estimate the synergistic toxicity of MPs and METH.

Therefore, the toxicity of individual PS and PVC MPs and the joint effects of MPs and METH on zebrafish larvae were investigated in this work. The aims were to (1) elucidate the adverse effects on larval developmental processes, (2) reveal the impacts on the locomotor activity of fishes, (3) demonstrate the histopathological alterations of the larvae, and (4) elucidate the molecular mechanisms. Through comprehensive analysis and exploration of the aforementioned aspects, it is possible to investigate whether the presence of MPs enhances or attenuates the toxicity of METH.

## 2. Materials and Methods

### 2.1. Animals and Experimental Design

Zebrafish (Danio rerio) embryos were obtained from Shanghai Fei Xi Biotechnology Co. (Shanghai, China) and incubated at the Department of Marine Biology, Hohai University, according to the animal research protocol approved by the Institutional Animal Care and Use Committee. Embryos were cultivated in embryo-rearing media (ERM; containing 1 g NaCl, 0.03 g KCl, 0.04 g CaCl_2_·2H_2_O, and 0.163 g MgSO_4_·7H_2_O in 1 L DD water, adjusted to pH 7.2) until hatching [41]. After hatching, the larvae were incubated in cultivation water with the following parameters: dissolved oxygen > 7.0 mg L^−1^; pH 7.0–7.4; and no detectable residual chlorine, ammonia, or nitrite. Water quality and environmental conditions were rigorously maintained, with a 14:10 light:dark photoperiod at 26 ± 1 °C. During cultivation, the parental zebrafish were fed twice daily with frozen brine shrimp (Artemia nauplii) from a fresh source. Each breeding group consisted of 6 male zebrafish and 3 female zebrafish. Spawning behavior was induced using light after a period of darkness, and, after 4 h of fertilization, the fertilized eggs were collected.

The standards PS and PVC MPs (average diameter: 10 μm) were purchased from Dongguan Junxin Plastics Co. Ltd., (Dongguan, China). The purity and characteristics were identified by a Thermo Scientific Nicolet iN10 infrared spectrometer (Thermo Scientific, Waltham, MA, USA). The distributions of particle size were determined using a Malvern mastersizer 2000 laser particle sizer (Malvern Ltd., Malvern, UK). Hydrochloride salts of METH were purchased from Cerilliant (Cerilliant, 1.0 mg mL^−1^ in methanol). The stock solution was diluted using ERM to prepare the working solution of METH for use.

**Figure 1:**
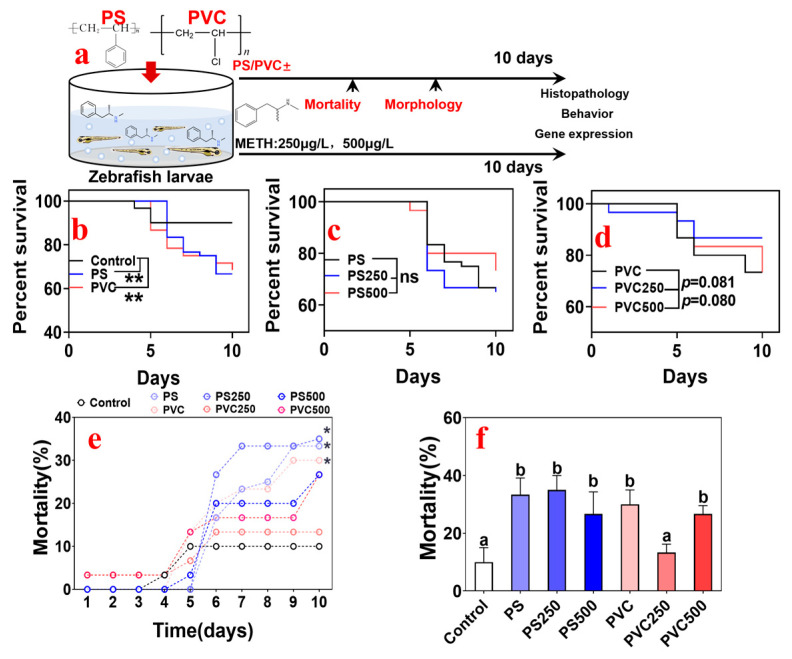
(**a**) The timeline of the experiments: zebrafish larvae (24 h post-fertilization) were exposed to PS MPs, PVC MPs, PS MPs + METH, and PVC MPs + METH for 10 days; during the exposure, the mortality and morphological abnormality of fish were recorded; at the end of the exposure, the histopathological, behavioral, and genetic biomarkers of fish were analyzed. (**b**–**d**) The survival curves of animals during the exposure in different groups. (**e**) The trajectory curves of mortality at different time points in different groups. (**f**) The terminal mortality of fish in different groups. * *p* < 0.05, ns > 0.05 vs. the control. Distinct letters denote statistically significant differences among the respective groupings.

Embryos at the early blastula stage (24 h post fertilization) were randomly placed in a fresh 60-mm crystallizing dish (20 embryos per dish) spiked with MPs or METH. The exposure lasted for 10 d [42]. Seven groups were set: CK group (ERM blank control); PS group (PS MPs at 20 mg L^−1^); PVC group (PVC MPs at 20 mg L^−1^); PS250 group (PS MPs 20 mg L^−1^ + METH 250 μg L^−1^); PS500 group PS MPs (20 mg L^−1^ + METH 500 μg L^−1^); PVC250 group (PVC MPs 20 mg L^−1^ + METH 250 μg L^−1^); PVC500 group (PVC MPs 20 mg L^−1^ + METH 500 μg L^−1^). Each group was replicated with three dishes, and 100% of the exposure solution was replaced every 24 h by transferring the animals into a new dish spiked with fresh exposure solution. All animal studies were performed in accordance with the Guidelines for Animal Experiments of Hohai University, which meet the ethical guidelines for experimental animals in China. To ensure that the dose of MPs was constant, the exposure solution was prepared by weighing in precision using an ultramicroanalytical balance (accurate to 0.1 μg) and used immediately afterward. The levels of METH in the dosing solution were confirmed using HPLC-MS/MS, as previously described [43]. The actual concentrations were 99.3–100.9% of the nominal values (Table S1), and the details are shown in the Supporting Information. During exposure, the mortality and morphology of the embryos were recorded daily using a stereomicroscope (Shang Guang, Suzhou Instrument Co., Ltd., Suzhou, China). At the end of the experiment, embryos were rinsed using fresh ERM three times, and the associated biomarkers were analyzed immediately. The timeline of this work is shown in Figure 1a.

### 2.2. High-Performance Liquid Chromatography Tandem Mass Spectrometry (HPLC-MS/MS) Detection Method

The 1 mL of medium spiked with METH was pipetted and then filtrated through 0.22 μm cellulose acetate membrane filters. The filtrate (300 μL) was added with an internal standard of METH-d8 with the ultimate dose at 200 ng. Subsequently, chemicals in the mixture were quantified using a UFLCXR-LC system (Shimadzu, Japan) with a Phenomenex Gemini C18 column (100 mm × 2 mm, 3 μm) and an injection volume of 5 μL. The mobile phase was composed of 30 mM ammonium formate in ultrapure water with pH adjusted to 3 using formic acid (98% in water) (A) and 0.1% formic acid in AcN (B). The flow rate of the mobile phase was controlled at 0.3 mL min^−1^. The elution gradient was as follows: 0–0.1 min: 5% B; 0.1–3.0 min: 30% B; 3.0–5.0 min: 80% B; 5.0–7.5 min: 90% B; 7.5–7.6 min: 5% B; 7.6–13.0 min: 5% B. Concentrations were determined using an API 4000 triple quadrupole mass spectrometer (AB SCIEX, MA, USA) equipped with an electrospray interface operating in positive ionization mode. The quantification of MS system was operated in multiple reaction monitoring (MRM) mode.

### 2.3. Behavioral Function Measurement

At the end of the exposure, eleven fish were collected randomly from each group and subsequently placed into a 6-well plate (one fish per well) spiked with 2.5 mL fresh ERM without MPs and METH. The test was conducted in the light photoperiod after acclimation for 20 min. The locomotion video of each fish was recorded using a CCD camera (Mingchuangda Co. Ltd., Shenzhen, China) for 5 min. After analysis of the baseline, 2 min of the video in which the fishes were at steady state was used to analyze the behavioral parameters (i.e., immobility duration, mean velocity, and turn angle) using ImageJ 1.53u software (National Institutes of Health, Bethesda, MD, USA) with the built-in analysis tool [44]. The immobility duration (s min^−1^) was defined as the proportion of the duration that the fish kept still of the total measured time. The mean velocity (cm s^−1^) was the distance traveled per unit time. The turn angle (degree) was the change in movement direction (clockwise or anti-clockwise).

### 2.4. Histological Examination

After exposure, five zebrafish larvae were randomly collected from each group, euthanized by rapid cooling, fixed in 4% paraformaldehyde (PFA) for more than 24 h, dehydrated in a graded ethanol series, and embedded in paraffin. Sections of the larvae were cut at 8 μm and stained with hematoxylin and eosin (H&E). The obtained slices were examined by a light microscope equipped with an ocular micrometer for alterations in histopathology. The rank evaluations on the histopathological changes of zebrafish were determined using the histopathological assessment tools provided previously [45]: Hemorrhage, hyperemia, and inflammation infiltration scored as 1; nuclear alterations and hyperplasia scored as 2; necrosis scored as 3; and malformation scored as 4.

### 2.5. Quantitative Real-Time RT-PCR Assay

After exposure, larvae (fifty individuals per sample, n = 3/group) were randomly selected to quantitatively analyze the transcriptional levels of genes, including *sod*, *cat*, *gpx1a*, *gpx4a*, *gstt1a*, *fosab*, *fosb*, *egr2a*, *egr2b*, *egr4*, *tnf-α*, *il-6*, *tp53*, *casp3*, *rrm2*, and *β-Actin*. Total RNA of each sample was extracted, reverse transcribed, and determined by real-time polymerase chain reaction. Detailed information is described in the Supporting Information, and all primer sequences are shown in Table S2.

### 2.6. Statistical Analysis

Data were analyzed using SPSS 26 software (SPSS, Inc., Chicago, IL, USA). The normality of the data was determined using Shapiro-Wilk’s test. Homogeneity of variance was assessed using Levine’s test. The survival curve was established by GraphPad Prism 8.0 (GraphPad Software, San Diego, CA, USA), and the differences were estimated using the log rank test. The differences in the mortality observed at different time points between the control group and the exposure groups were determined using two-way ANOVA. One-way ANOVA followed by Tukey’s test was used to determine the differences in the terminal mortalities, values of behavioral parameters, and transcriptional levels of genes among different groups. Student’s t test was used to analyze the differences in the data between two groups. The correlation analysis among different parameters of fish in different groups was conducted by calculating Pearson’s correlation coefficients. All data are expressed as the mean ± standard deviation (SD). Statistical significance was considered as *p* values less than 0.05 (95% confidence interval).

## 3. Results

### 3.1. Characterization Map, Zeta Potential, and Analysis of MPs

Monomers of PS and PVC MPs appeared as smooth and intact spheres (Figure S1). The average sizes of PS and PVC used in this work were 6.998 and 3.760 μm, respectively. As listed in Table S3, the zeta potentials of PS and PVC MPs were ± 45 and ± 20 (pH = 7 and 25 °C), respectively.

### 3.2. Effects of Combined MPs and METH Exposure on the Mortality of Zebrafish Larvae

During the 10 d exposure, the death of larvae in the control group occurred until 5 d, while exposure to individual PS or PVC induced constant wastage of the community (*p* < 0.01 for PS and PVC, Figure 1b). Compared with the individual exposure groups, there were no significant differences observed in the joint exposure groups (Figure 1c,d). Considering the effect of exposure duration, the daily changes in mortality from different groups were analyzed (see Figure 1e). The slope of the changing curve of mortality of the PS groups was greater than that of the PVC group. According to the terminal lethal results (Figure 1f), the total mortality of MPs combined with METH was lower than that of single MPs exposure and markedly greater than that of the control. Meanwhile, there was a significant difference in total mortality between the PVC250 and PS250 groups (*p* < 0.01).

### 3.3. Effects of Combined MPs and METH Exposure on the Locomotor Behavior of Zebrafish Larvae

**Figure 2:**
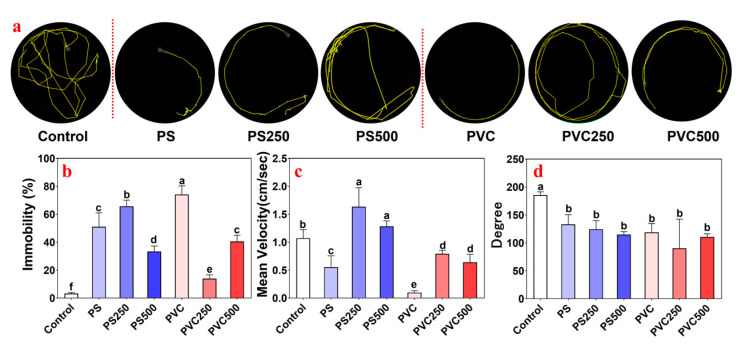
Behavioral changes of zebrafish larvae in different groups after 10-day exposure. (**a**) Representative swimming trajectories of zebrafish larvae. (**b**–**d**) The mobility rate, mean velocity, and degree of zebrafish larvae of the group exposed to PS MPs, PS MPs + METH, PVC MPs and PVC MPs + METH. Distinct letters denote statistically significant differences among the respective groupings.

Unexposed zebrafish exhibited a characteristic behavior of swimming along the walls of the container (the control in Figure 2a). Notably, treatment with MPs or METH markedly induced an abnormal behavioral phenotype in fishes, characterized as locomotor activity syndrome with lateralized circling behaviors and postural asymmetry. As shown in Figure 2a, the animals from the PS group and PVC group showed decreased swimming activity compared to the control, while the addition of METH alleviated this effect. The immobility duration of larvae exposed to individual PS and the mixture of PS and METH increased significantly compared with the control (*p* < 0.05), featuring a reverse “U” shape (Figure 2b). The peak was observed in the PS250 group. For the mean velocity (Figure 2c), individual exposure to PS reduced motility, but joint exposure to PS and METH at 250 μg L^−1^ strikingly stimulated movement and then returned to normal levels (the control) with METH up to 500 μg L^−1^. The changes in the turn angle of fishes from the PS group showed a concentration-dependent manner (Figure 2d). Notably, the immobility duration of zebrafish from the PVC group showed the opposite trajectory as a “U” shape, in which the culmination was observed from the individual PVC group (Figure 2b). Treatment with PVC markedly suppressed the locomotion of fishes (*p* < 0.05), while the values increased with METH addition (Figure 2c). Meanwhile, the alterations in the turn angle from the PVC groups showed a similar pattern to the PS group (Figure 2d).

### 3.4. Histopathological Effects of Combined MPs and METH Exposure on Zebrafish Larvae

**Figure 3:**
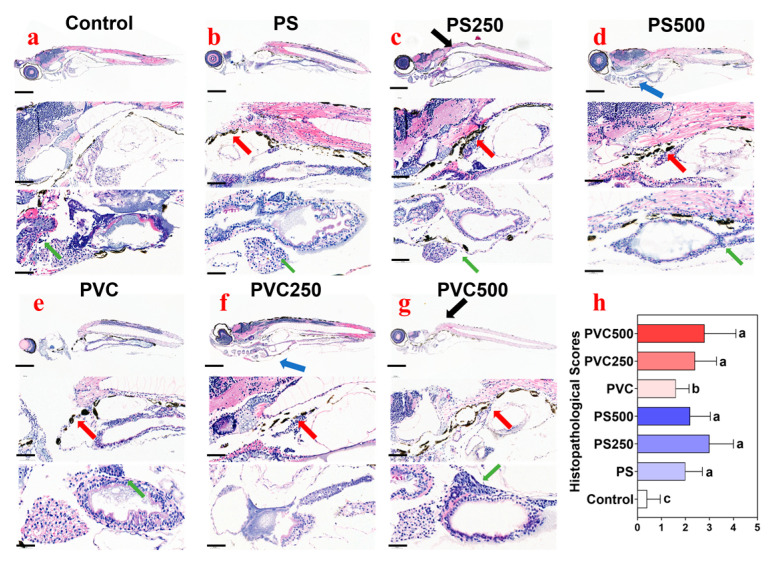
Histopathological changes of zebrafish larvae observed in the control group (**a**), PS MPs group (**b**), PS MPs + METH (**c**,**d**), PVC MPs (**e**), and PVC MPs + METH (**f**,**g**), as well as the histopathological scores in different groups (**h**). Pleural effusion (blue arrow), cyrtosis (black arrow), inflammatory cell infiltration (green arrow) and intestinal contents (red arrows). Scale bar = 100 μm. The rank evaluations on the histopathological changes of zebrafish (**h**). Distinct letters denote statistically significant differences among the respective groupings.

Obvious particulate-like sediments were found in the enteric cavity of fish from the exposure groups (red arrow) (Figure 3b–g) compared with the control (Figure 3a), suggesting that MPs are foraged by larvae through the digestive tract. Meanwhile, a variety of histopathological changes were observed in the fish from different exposure groups, including cyrtosis (black arrow), pleural effusion (blue arrow), and inflammatory cell infiltration (green arrow) (Figure 3b–g) in comparison with the control (Figure 3a). According to the histopathological score evaluation (Figure 3h), combined exposure to PVC MPs and METH at 500 μg L^−1^ induced the most significant changes, followed by PS MPs + METH at 250 μg L^−1^, and PS MPs showed slightly greater effects than PVC MPs.

### 3.5. Effect of Combined MPs and METH Exposure on Gene Expression in Zebrafish Larvae

**Figure 4:**
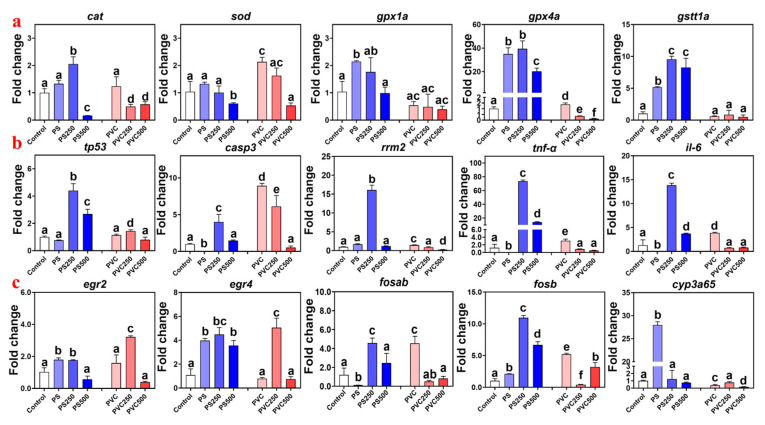
Relative gene expression levels of zebrafish larvae in different groups involved into the pathways including oxidative stress pathways (**a**), apoptosis (**b**), and xenobiotic metabolism (**c**). All the data were expressed as mean ± S.D. Distinct letters denote statistically significant differences among the respective groupings.

The relative expression levels of the *sod*, *cat*, *gpx1a*, *gpx4a*, and *gstt1a* genes involved in oxidative stress were determined in this work (Figure 4a). Individual exposure to MPs generally significantly induced the expression of antioxidant enzyme-related genes. The fold changes were 2.14-fold to 34.95-fold, respectively. After the addition of METH, their relative expression levels showed a significant decrease (−0.89-fold to 14.75-fold).

The relative expression levels of the *tnf-α*, *il-6*, *casp3*, *rrm2*, and *tp53* genes involved in the p53 signaling pathway responsible for apoptosis were determined in this work (Figure 4b). The exposure of zebrafish larvae to MPS and the blend of MPs and METH resulted in an inverted “U” shaped change in the expression levels of immune-related genes, with the maximum value observed in the PS250 group. The fold changes were 4.40-fold to 73.53-fold, respectively. In the PVC exposure group, the crest was observed in the individual PVC exposure group (1.13-fold for tp53, 8.92-fold for *casp3*, 1.46-fold for *rrm2*, 3.09-fold for *tnf-α*, and 3.87-fold for *il-6*).

Given the pronounced histopathological changes found in fish from the exposure groups, the mRNA transcriptional levels of larvae development- and metabolism-associated genes (i.e., *egr2*, *egr4*, *fosab*, *fosb*, and *cyp3a65*) were analyzed. As shown in Figure 4c, all the changes in PS MPs groups showed the reverse “U” shape, with the maximum values (except *cyp3a65*) found in the PS250 groups, and the fold-change ranged from 1.76-fold to 107.84-fold. For *cyp3a65*, the peak was observed in the PS group (30-fold). In the PVC groups, the transcriptional levels of the genes *egr2*, *egr4*, *fosab*, and *fosb* were upregulated 3.22-fold to 5.20-fold, but *cyp3a65* was downregulated by 0.16-fold.

### 3.6. Correlation Analysis between the Different Biomarkers of Fish in Different Groups

**Figure 5:**
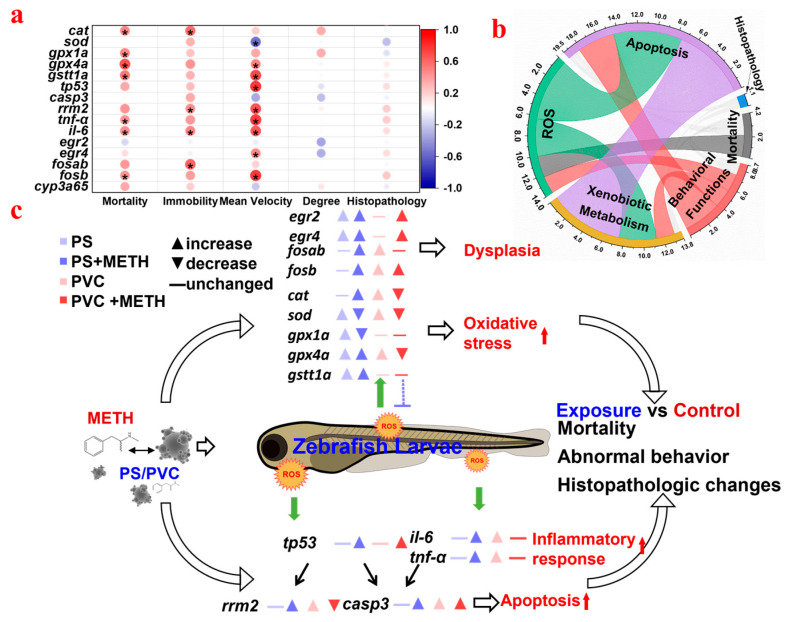
The interaction relationships among lethal effects, behavioral disorders, histopathological damage, and genetic expression changes (Pearson’s correlation coefficients) (**a**); a chord diagram is employed to visualize and elucidate the cross−talk among various parameters (**b**); a pattern diagram depicting alterations in gene expression profiles in zebrafish larvae following the ingestion of polypropylene microplastics (PP MPs) and methamphetamine in vivo (**c**). * *p* < 0.05.

To further elucidate the interaction relationships among lethal effects, behavioral disorders, histopathological damage, and genetic expression changes, Pearson’s correlation coefficients were calculated with the genes on the *y*-axis and physiological indicators on the *x*-axis (Figure 5a). Mortality was positively correlated with most of the genes, and immobility was positively related to the genes *cat*, *rrm2*, *il-6*, and *fosb* (*p* < 0.05). Mean velocity showed covariation relationships with the genes involved in oxidative stress and the P53 signaling pathway. Furthermore, a chord diagram was established to illuminate the cross-talk among different parameters (Figure 5b). ROS were strongly linked to apoptosis and xenobiotic metabolism and moderately associated with behavioral functions and mortality, indicating that they might be the primary events for the adverse outcomes in zebrafish posed by MPs and METH. Otherwise, apoptosis and xenobiotic metabolism showed a correlative relationship. Histopathological changes were weakly associated with apoptosis, behavioral functions, and xenobiotic metabolism.

## 4. Discussion

### 4.1. The Effects on the Ecological Function Associated Indicators of Zebrafish Larvae by MPs

Much existing evidence, such as aberrant swimming behavior, glycolipid metabolism disorder, and immune responses, has revealed the toxicity of PS to fish [9,10,11,12]. The MPs utilized in this study have a diameter of 10 μm. Previous research by Lin et al. indicated that they are unable to enter the chorionic pore of zebrafish embryos [46]. However, it is widely acknowledged that MPs adsorbed on the surface of the chorionic pore can still exert an impact on zebrafish embryos (e.g., increase in incubation time and increased heart rate) [47,48]. After hatching of zebrafish larvae, MPs enter the larvae by being mistaken for food or carried into the larvae by water, thus causing toxicity in behavior and development [10]. Similarly, we proved the toxicity of 20 mg L^−1^ PS on the behavior and development of zebrafish larvae, indicating that ecological risks should be considered (Figure 5c). Parallel to previous assessments on the toxicity for zebrafish embryos induced by PVC MPs [29], intestinal damage and oxidative stress in the animals were observed. Otherwise, the increase in mortality and associated deformations were first reported in this work. Considering the cocktails of MPs and emerging organic pollutants in aquatic systems, assessment of synergetic toxicity is imperative. Since the adsorption of METH by MPs was evidenced [40], the different toxicological effects posed by individual PS and PVC MPs and joints with METH were investigated.

### 4.2. Oxidative Stress

Fish, especially during their early life stages, are vulnerable to oxidative stress [49]. Recent research has emphasized that exposure to MPs is a major contributor to oxidative stress in zebrafish [50]. This study specifically investigated the impact of MPs on oxidative stress in zebrafish larvae. The results demonstrated that a single exposure to PS MPs significantly influenced oxidative stress by upregulating the expression of the *gpx1a*, *gpx4a*, and cat genes. These genes play a critical role in converting hydrogen peroxide into water, thereby maintaining cellular oxidative balance [29]. Notably, single exposure to PS MPs did not significantly induce *sod* expression, whereas PVC exposure led to a significant increase in *sod* expression but did not affect cat and *gpx1a* expression. This suggests that the impact of MPs on oxidative stress is contingent upon the type of MPs present, with PS MPs inducing higher levels of oxidative stress than PVC MPs. This study revealed that the addition of METH resulted in a significant reduction in the expression of antioxidant enzyme genes in zebrafish larvae, particularly at a concentration of 500 μg L^−1^ METH. The results of the correlation analysis confirmed a strong positive correlation between the expression of antioxidant enzymes and the mortality rate (Figure 5a). This implies that oxidative stress is the main cause of mortality in zebrafish larvae, and METH and MPs exhibit an antagonistic relationship in terms of oxidative stress and lethal toxicity. These findings shed light on the intricate interactions between MPs and substances like METH in influencing oxidative stress responses and mortality in zebrafish larvae. 

Oxidative stress is closely related to inflammatory reactions [51]. Due to the ROS induced by MPs exposure, the p53 pathway has been shown to be significantly upregulated, leading to cell apoptosis and DNA damage [52]. The histopathological examination of zebrafish larvae showed a significant inflammatory reaction, including inflammatory cell infiltration and pleural edema. Meanwhile, suspected levels of MPs were also found in the digestive tract, which have been confirmed to cause mucosal damage and inflammation [53]. Genes *il-6* and *tnf- α* are inflammatory factors, and their relative expression can represent the degree to which the body is affected by inflammation Interestingly, we found that exposure to PVC significantly up-regulated the expression of associated genes, while there were no marked changes observed after exposure to PS alone. Addition with METH mited the changes for PVC but catalyzed the effects for PS. At the same time, the inflammatory response also activated the p53 pathway related to cell apoptosis. By examining downstream genes [54], including *tp53*, *casp3*, and *rrm2*, a positive correlation was revealed between their expression levels and the expression level of inflammation-related genes. The significant increase in *casp3* expression indicates the induction of apoptosis, leading to the death of zebrafish larvae [55]. In addition, changes in *rrm2* expression indicate DNA damage, as organisms attempt to regulate and repair damaged DNA [56]. The potential reasons may be that the adsorption capacity of PS and polar organic pollutants is significantly higher than that of PVC [30]. Meanwhile, the zeta potential of PVC determines that its alternating stability in water is not as good as that of PS, and it is more prone to agglomeration, thereby reducing the adsorption of METH [57]. As a result, PS might carry much more METH into the zebrafish body than PVC, and it leads to more severe oxidative stress phenomena. More experiments should be conducted in the future to test this hypothesis. These findings highlight the potential synergistic effects of MPs and emerging organic pollutants in aquatic environments and the need for continued research to better understand their mechanisms of toxicity and ecological impacts.

### 4.3. Ecological Implications

Behavioral functions are important indicators for assessing the neurotoxicity of pollutants on aquatic organisms, including fishes. Changes in normal behavior can have significant consequences at the ecosystem level [58]. Swimming ability, for instance, directly interferes with the success of fish in avoiding predators and actively feeding [59]. In this study, zebrafish larvae exposed to MPs alone showed an aberrant swimming trajectory compared with the control, which was consistent with the results found in a previous publication [10]. When exposed to a combination of MPs and METH, a hormetic response was observed, with stronger effects at a concentration of 250 μg L^−1^ and alleviated effects at 500 μg L^−1^. The observed phenomenon may be attributed to the primary stimulatory effects of METH at low concentrations and counteractive effects at higher doses. In line with findings in existing literature, *Oryzias latipes* display a significant increase in movement at lower concentrations of METH exposure. However, beyond a certain threshold concentration, this heightened movement begins to decrease, aligning with the results of our study. The potential reason for this change in our study could be the variation in METH adsorption capacity among different types of microplastics, resulting in differences in the concentrations to which zebrafish larvae are exposed [43]. Furthermore, the turn angle, which represents the behavior of exploring the environment, decreased in a concentration-dependent manner with exposure to MPs and METH, indicating an increased risk of predation for the exposed fishes [58]. Exiting studies have revealed that zebrafish exhibit reduced activity accompanied by inflammatory responses [60]. Similarly, the results of correlation analysis showed a strong correlation between inflammation-related genes and immobility, which may be due to the “anxiety” phenomenon induced by inflammation in zebrafish larvae [61]. In addition, oxidative stress-related genes also exhibited covariation relationships with behavioral changes in zebrafish larvae. Chen et al. suggested that oxidative stress could affect brain neurodevelopment, thus influencing the behavioral changes in zebrafish larvae [62], which might be one of the reasons for the abnormal swimming speed observed in zebrafish larvae.

In addition, MPs may interfere with the early development of zebrafish larvae, thereby impairing their motor ability [10], especially the neurodevelopmental toxicity [63] caused by them. In this study, the relative expression of genes related to the early development of zebrafish larvae was also studied; among these genes, *fosab* and *fosb* are translation factors in zebrafish brain nerves that act as natural markers and participate in early embryonic development [64]. In this study, the amount of gene expression was also the same as the type of MPs. In addition, there were diametrically opposite changes after adding METH. Such abnormal expression may lead to abnormal brain development and consequently affect the response of zebrafish larvae to external stimuli. In addition, early growth response (egr) factors have been elucidated to be related to the proliferation and differentiation of zebrafish in early life [65]. Recent studies by Lee et al. have shown that exposure to PS MPs significantly downregulates the expression of mouse EGR family genes [66]. Consistent with these findings, in this study, similar changes in *egr* factor expression were observed in zebrafish exposed to PS and PVC MPs. However, the addition of METH alleviated the adverse effects on *egr* factors, indicating a potential beneficial effect of METH combination therapy in alleviating the developmental delay caused by MPs. In particular, *egr2* has been found to regulate the expression of *cyp26b1* through retinoic acid signaling, and *cyp26b1*, which is mainly expressed in osteoblasts, has been proven to cause head and face defects and axial bone abnormalities in zebrafish [67,68]. Therefore, the interference of PS and PVC MPs on the functions of the *egr* factor and *c-fos* factor in zebrafish larvae may be the cause of movement and morphological changes in the exposed group. Further research is needed to fully understand the potential mechanism of interaction between the expression of MPs, METH, and key regulators in zebrafish larvae.

### 4.4. Perspectives for Joint Toxicity of METH and PS/PVC MPs for Aquatic Organisms

Overall, the study indicates that PS and PVC MPs have distinct toxic effects on zebrafish larvae when exposed individually. PS MPs exposure significantly induces the expression of *cat*, *gpx1a*, and *gpx4a*, which are related to oxidative stress, while PVC MPs exposure mainly leads to a significant induction of *sod*. This suggests that PS exposure results in a more pronounced increase in oxidative stress-related enzymes compared to PVC exposure, and the expression of these oxidative stress-related enzymes is significantly correlated with the mortality rate. Moreover, when combined with METH, joint exposure to PS MPs and METH activates the p53 signaling pathway and enhances the expression of inflammation-related factors. In contrast, combined exposure to PVC MPs and METH inhibited their expression. These findings may be attributed to the ability of MPs to carry METH and the inherent differences in the toxicity of MPs themselves. It is worth noting that even though both types of MPs are categorized as <10 microns in size, there may be variations in particle size between PS and PVC MPs, potentially contributing to differences in their toxicity based on previous research [69]. In conclusion, further research involving joint toxicity experiments on MPs and psychotropic drugs is warranted to gain a better understanding of the intricate relationship between MPs and psychotropic drugs. Such investigations will help uncover the mechanisms underlying their combined effects and shed light on the potential ecological implications of these interactions.

## 5. Conclusions

In summary, the characteristics of the synergetic toxicity of MPs and METH was highly dependent on the type of MPs. Individual exposure to PS was more toxic than PVC based on the changes of the mortality and the behavioral functions. Meanwhile, joint exposure by PS and PVC with METH featured varying patterns, which might attribute to the different adsorption capacity of this two MPs. The cocktail of PS and METH was more likely to pose adverse effects on zebrafish larvae. This study, for the first time, estimated the different toxicity between PS and PVC MPs and the joint effects posed by MPs and METH. The results demonstrated the necessary for long-term monitoring of the pollutions in aquatic environments.

## Data Availability

Data are contained within the article.

## Supplementary Materials

The following supporting information can be downloaded at: https://www.mdpi.com/article/10.3390/toxics12010009/s1, Figure S1: Electron microscope scans of PS and PVC microplastics, Monomer of PS (a–c) and PVC (d–f) MPs showed as smooth and intact sphere; Table S1: The measured and nominal concentrations of METH in exposure solution; Table S2: Primer sequences for the genes tested in the present study; Table S3: ZETA potential of PS and PVC microplastics (pH = 7, 25 °C); Table S4: Relative expression change fold of genes in PS and METH exposure combination. Table S5: Relative expression change fold of genes in PVC and METH exposure combination.
