# Supplementary material for: Methamphetamine Shows Different Joint Toxicity for Different Types of Microplastics on Zebrafish Larvae by Mediating Oxidative Stress
Source: Toxics. 2023 Dec 22;12(1):9. doi: 10.3390/toxics12010009 (PMC10819112; doi:10.3390/toxics12010009)
Supplement: Supplementary file 1 [file toxics-12-00009-s001.zip › toxics-2771171-supplementary.pdf]

Supporting Information

for

Methamphetamine Shows Different Joint Toxicity for Different Types of Microplastics on Zebrafish Larvae by Mediating Oxidative Stress

Jindong Xu [1], Wenqi Yang [1], Dongyi Wang [1], Zhenglu Wang [2], Chuang Liu [1] and Jiana Li [3,*]

[1] College of Oceanography, Hohai University, Nanjing 210098, China; aukuma20@gmail.com (J.X.); eliomurphy8@gmail.com (W.Y.); wdynn@foxmail.com (D.W.); huangliu2020@hhu.edu.cn (C.L.)
[2] West China School of Public Health and West China Fourth Hospital, Sichuan University, Chengdu 610041, China; m18018567955@163.com
[3] Ningbo Academy of Ecological and Environmental Sciences, Ningbo 315000, China
* Correspondence: stlijiana@163.com; Tel.: +86-185-1258-1998

Table S1 The measured and nominal concentrations of METH in exposure solution.

Nominal concentration	Actual concentration	Relative percentage %
250 μg L-1 in PS group	248.3 ± 3.2 μg/L	99.3
500μg L-1 in PS group	500.2 ± 7.8 μg/L	100.0
250 μg L-1 in PVC group	252.3 ± 5.7 μg/L	100.9
500μg L-1 in PVC group	499.3 ± 10.2 μg/L	99.9

Table S2. Primer sequences for the genes tested in the present study

Genes name	Primer name	Primer sequence (5’-3’)	Genbank No.
lipca	Forward	ACTGAGCCTGAAGCCAAGATGAAG	NM_201022.1
	Reverse	CGTCTACCGACCAGCCATGAATG	
pklr	Forward	AACACAGATGCTGGAGAGTATG	NM_201289.1
	Reverse	CTCTACAGGGAAGTGTCCTTTG	
cyp3a65	Forward	TTCTACGCCGCCTTACAGAAG	NM_001037438.1
	Reverse	GGTCACTCAGACCTTTCTCCG	
fosab	Forward	TTACCACCTTACCCCAC	NM_205569.1
	Reverse	TGGACCATCCACTGCAAGTC	
fosb	Forward	ATGATGCAGGAGAGGGAGCCT	NM_001328202.1
	Reverse	TGTGCTGTTTTCGATAGGCAAGC	
egr2a	Forward	TCTGGATGAGATTCCCCCGT	NM_001328404.2
	Reverse	CTGCTCAGGCCACCTGC	
egr2b	Forward	AGTGCTTCTTAGGACTTCACGA	NM_130997.2
	Reverse	CCGTTAATCAGGCCATCTCC	
egr4	Forward	AGACGGACTCGGTTTTGGAC	NM_001114453.1
	Reverse	ATTGCCCTGGGTCTTATGG	
tnf-α	Forward	AAGGAGAGTTGCCTTTACCG	NM_212859.2
	Reverse	ATTGCCCTGGGTCTTATGG	
il-6	Forward	ATGCCATCCGCTCAGAAAACAG	NM_001261449.1
	Reverse	CACATCCTGAACTTCGTCTCCA	
casp3	Forward	CCGCTGCTCACTA	NM_131877.3
	Reverse	ATCCTTTCACGACCATCT	
tp53	Forward	CAAAGCAATGGCGCAAAACG	NM_001271820.1
	Reverse	CCGGGATAGTCGCTTGTCTC	
cat	Forward	GCATGTTGGAAAGACGACAC	NM_130912.2
	Reverse	GCCCACATAGAAATGCACAG	
sod	Forward	GGGTGGCAATGAGGAAAG	NM_131294.1
	Reverse	GCCCACATAGAAATGCACAG	
gpx1a	Forward	AAAATGTGGCGTCGCTTTGAG	NM_001007281.2
	Reverse	ATTCTTGCAGTTCTCCTGGTGC	
gpx4a	Forward	CCGTGAAGGCCAGGTTG	NM_001007282.2
	Reverse	GCCCTCAGGAGGAGGTG	
gstt1a	Forward	GGTCTTCTGGTTCAAGGGAG	NM_001327762.1
	Reverse	GGTCTTCTGGTTCAAGGGAG	
rrm2	Forward	ATTGAGTTTGTGGCTGACCG	NM_131450.3
	Reverse	GCCCTCCAACGAAATGTTC	

Table S3. ZETA potential of PS and PVC microplastics (pH=7, 25°C)

item	ZETA potential
PS	-52.91 ± 2.66 mV
PVC	-22.15 ± 0.16 mV

Table S4. Relative expression change fold of genes in PS and METH exposure combination.

Genes name	Change Fold							
	Control	SD	PS	SD	PS250	SD	PS500	SD
cyp3a65	1	0.07	27.99	0.56	1.25	0.97	0.74	0.07
fosab	1	0.59	0.11	0.004	4.58	0.42	2.46	0.82
fosb	1	0.26	2.11	0.02	10.95	0.28	6.68	0.41
egr2	1	0.21	1.79	0.09	1.765	0.03	0.56	0.21
egr4	1	0.42	3.98	0.14	4.48	0.47	3.56	0.33
tnf-α	1	0.71	0.003	0.004	73.53	1.50	14.29	0.44
il-6	1	0.91	0.006	0.004	13.84	0.35	3.70	0.09
casp3	1	0.05	0.01	0	4.01	0.83	1.46	0.07
tp53	1	0.05	0.77	0.01	4.40	0.41	2.69	0.28
cat	1	0.12	1.33	0.09	2.05	0.21	0.16	0.004
sod	1	0.30	1.32	0.05	1.01	0.19	0.60	0.02
gpx1a	1	0.30	2.14	0.02	1.76	0.42	0.99	0.17
gpx4a	1	0.13	34.95	4.33	39.4	5.49	20.20	2.27
gstt1a	1	0.23	5.17	0.07	9.55	0.39	8.26	1.16
Rrm2	1	0.05	1.68	0.12	16.10	1.04	1.2	0.07

Table S5. Relative expression change fold of genes in PVC and METH exposure combination.

Genes name	Change Fold							
	Control	SD	PVC	SD	PVC250	SD	PVC500	SD
cyp3a65	1	0.07	0.39	0.08	0.77	0.09	0.16	0.03
fosab	1	0.59	4.55	0.60	0.51	0.07	0.83	0.17
fosb	1	0.32	5.2	0.18	0.41	0.05	3.21	0.68
egr2	1	0.26	1.58	0.41	3.22	0.053	0.39	0.04
egr4	1	0.52	0.79	0.06	5.06	0.64	0.75	0.17
tnf-α	1	0.71	3.09	0.34	0.87	0.06	0.53	0.06
il-6	1	0.1	3.87	0.10	0.75	0.03	0.8	0.09
casp3	1	0.05	8.92	0.26	6.11	1.21	0.51	0.17
tp53	1	0.05	1.13	0.06	1.43	0.07	0.80	0.15
cat	1	0.12	1.24	0.28	0.5	0.006	0.58	0.07
sod	1	0.30	2.13	0.13	1.62	0.23	0.53	0.07
gpx1a	1	0.30	0.54	0.11	0.48	0.37	0.39	0.09
gpx4a	1	0.13	1.38	0.10	0.35	0.02	0.11	0.03
gstt1a	1	0.23	0.61	0.04	0.86	0.55	0.5	0.24
Rrm2	1	0.05	1.46	0.07	0.88	0.12	0.24	0.05

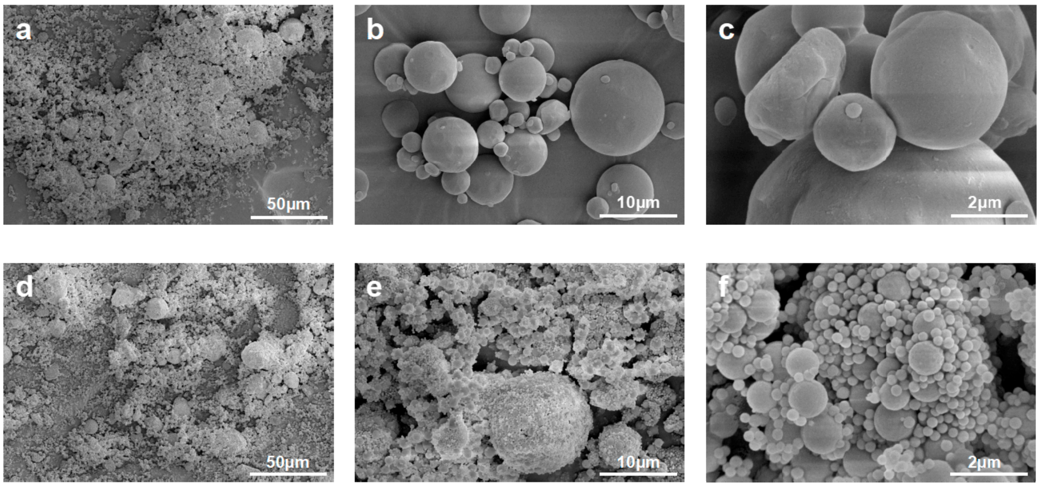

Figure S1. Electron microscope scans of PS and PVC microplastics, Monomer of PS (a-c) and PVC (d-f) MPs showed as smooth and intact sphere.
